# Neonatal goiter secondary to maternal iodine exposure

**DOI:** 10.1210/jcemcr/luag280

**Published:** 2026-10-01

**Authors:** Mohd Rafi Mir, Md Ejaz Alam, Basharat Q Dar

**Affiliations:** Department of Endocrinology, Government Medical College, Srinagar 190010, India; Department of Endocrinology, Government Medical College, Srinagar 190010, India; Department of Endocrinology, Government Medical College, Srinagar 190010, India

**Keywords:** goiter, iodine exposure, pregnancy

## Image legend

A term neonate born by normal vaginal delivery presented at birth with cyanosis and a visibly enlarged anterior neck swelling (Fig. 1). The mother had no known history of thyroid disease or exposure to antithyroid drugs (thionamides) during pregnancy; however, retrospective questioning revealed that she had taken an over-the-counter cough syrup suspected to contain iodine multiple times during pregnancy, although she could not recall the exact dosage. Physical examination revealed a diffuse enlargement of the thyroid gland. Thyroid function tests demonstrated severe primary hypothyroidism with TSH > 49.5 µIU/mL (reference range, 0.5-6.5 µIU/mL), total T4 3.16 µg/dL )40.7 nmol/L; reference range, 4-13 µg/dL [SI: 52-167 nmol/L]), and free thyroxine 0.5 ng/dL (6.4 pmol/L; reference range, 0.61-1.12 ng/dL [7.9-14.6 pmol/L]). Neck ultrasonography showed diffuse bilateral enlargement of the thyroid lobes with increased vascularity, consistent with neonatal goiter. The most likely mechanism is iodine-induced fetal hypothyroidism due to the persistent Wolff–Chaikoff effect, to which neonates are particularly susceptible because of their immature ability to escape from iodine-induced inhibition of thyroid hormone synthesis. Recognition of maternal iodine exposure is essential, as prompt diagnosis and early levothyroxine therapy can prevent irreversible neurodevelopmental impairment [1].

**Figure 1:**
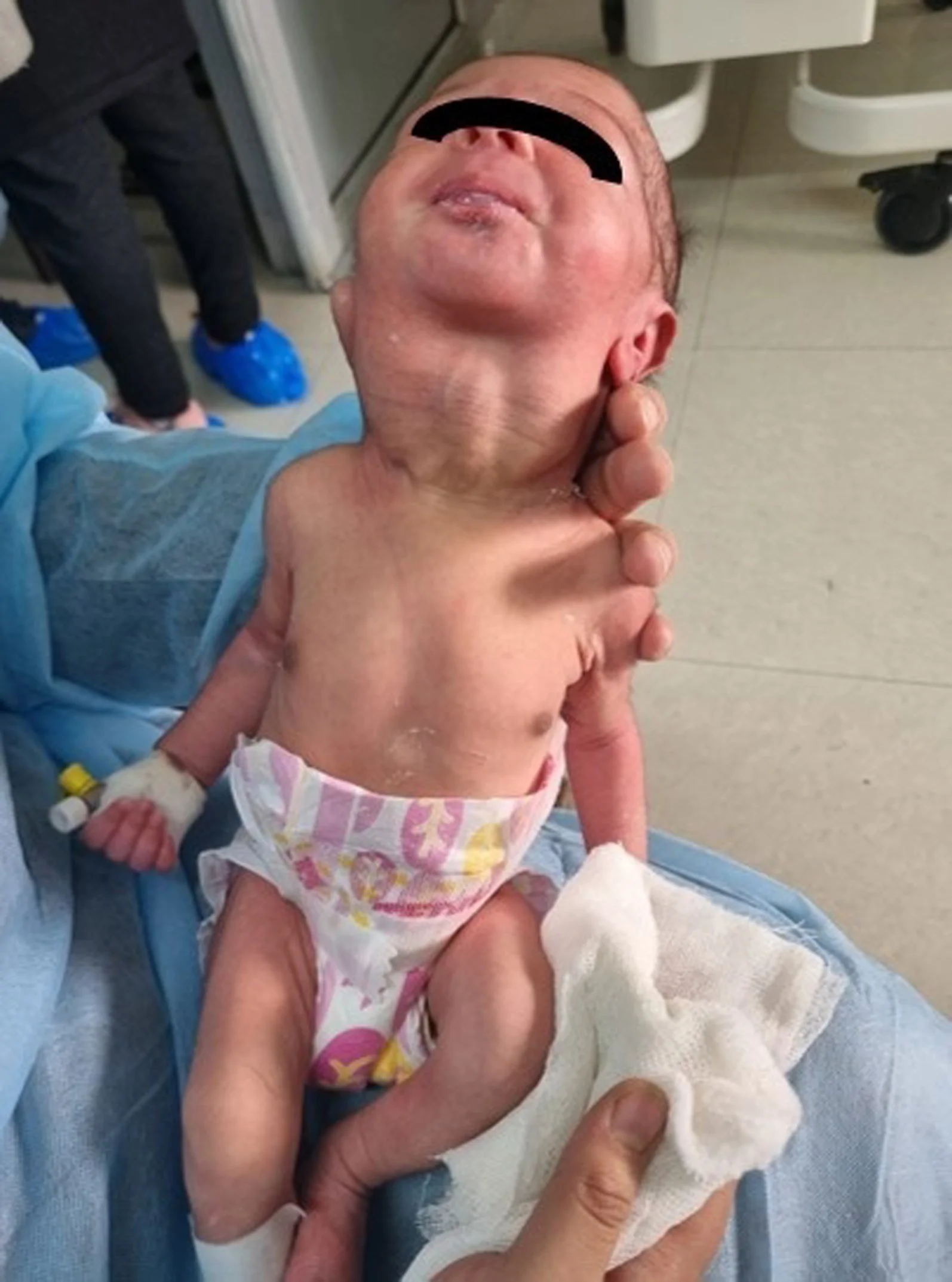
Neonatal goiter secondary to maternal iodine exposure.

## Contributors

All authors made individual contributions to authorship. M.R.M. and B.Q.D. were involved in the clinical diagnosis, workup, and management of the patient. M.E.A. was involved in data collection, literature review, and drafting the initial manuscript. All authors critically reviewed, edited, and approved the final version to be published.
